# Commentary: Noradrenaline and Dopamine Neurons in the Reward/Effort Trade-off: A Direct Electrophysiological Comparison in Behaving Monkeys

**DOI:** 10.3389/fnbeh.2015.00310

**Published:** 2015-11-16

**Authors:** Yuen-Siang Ang, Sanjay Manohar, Matthew A. J. Apps

**Affiliations:** ^1^Nuffield Department of Clinical Neurosciences, University of OxfordOxford, UK; ^2^Department of Experimental Psychology, University of OxfordOxford, UK

**Keywords:** dopamine, noradrenaline, motivation, decision, effort, reward, action, motor precision

A fundamental aspect of motivation is the evaluation of the costs and benefits of acting. When behavior is effortful, effort costs are weighed against expected rewards and behaviors that have sufficient value are selected. Once chosen, actions must also be sufficiently energized in order that rewards are obtained. Failures to appropriately motivate or energize behaviors are common and highly debilitating symptoms of many psychiatric and neurological disorders, and can significantly affect patients' quality of life (Barone et al., 2009). To ameliorate the negative effects of reduced motivation, a mechanistic understanding of the neurobiology of cost-benefit evaluation and energization is essential. Past research has implicated both dopamine (DA; Salamone and Correa, 2012) and noradrenaline (NA; Bouret and Richmond, 2015) as key neuromodulators in motivating and energizing behaviors. But, do these two neuromodulators play distinct roles in these processes?

In a recent study, Varazzani et al. (2015) conducted single neuron recordings of the substantia nigra pars compacta (SNc) and locus coeruleus (LC) while monkeys declined or accepted offers. On each trial, an offered reward could be obtained if a bar was squeezed at an offered level of force (3 reward sizes × 3 effort levels). The investigators found that at the times of *cue*s, which indicated the offered reward and effort, both SNc and LC activity were modulated positively by reward size. However, *only* SNc activity correlated negatively with the offered effort level. This led them to suggest that DA in the SNc may be involved in discounting reward value by effort, evaluating the option for choice selection. In contrast, around *action*-onset, although activity in both nuclei correlated positively with the required effort level, LC activity was modulated by effort significantly more than SNc. Strikingly, LC activity predicted force production and pupil dilatation even after factoring out the required effort level. Such effects were not observed in SNc neurons, indicating that the association between action-related activity and physical-physiological measures was particular to LC neurons.

Importantly, neurons in SNc are putatively dopaminergic, but neurons in the LC are predominantly noradrenergic. Thus, the researchers concluded that these two neuromodulators have distinct roles in motivation: whilst DA evaluates the choice of whether or not to exert effort, NA mobilizes resources necessary for exertion of effort *during* the execution of an action (Figure 1).

**Figure 1:**
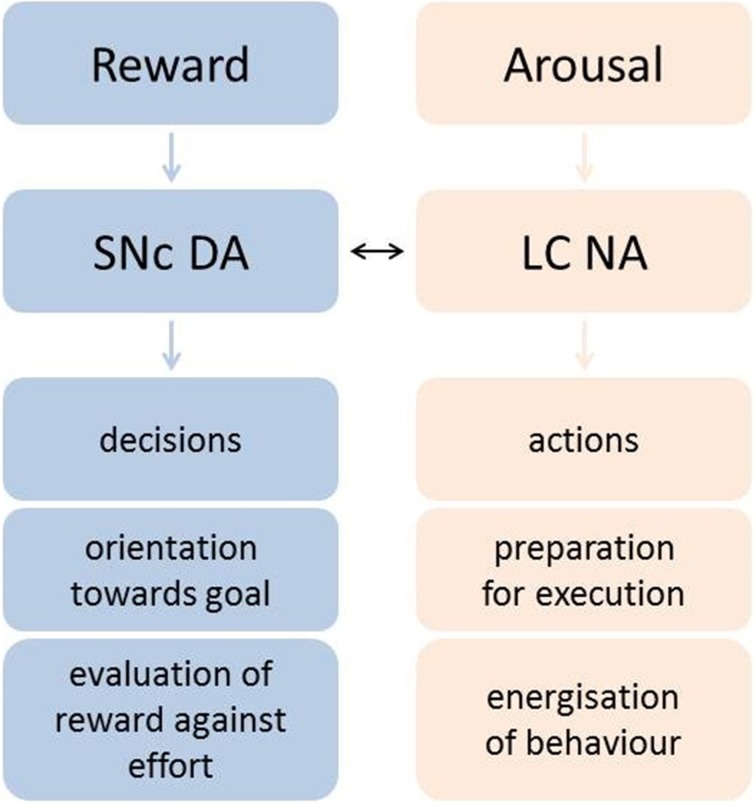
**Varazzani and colleagues' proposed roles of SNc DA and LC NA in motivation and goal-directed behavior**. DA signals net outcome value, orienting the organism toward the goal during the decision phase, whereas NA recruits resources to prepare and energize action.

One previous study also examined the relationship between SNc activity and cost-benefit valuation in a similar design (Pasquereau and Turner, 2013). Both studies demonstrated encoding of reward in SNc. Varazzani and colleagues also showed that overall spiking activity was reduced by effort cues, whereas Pasquereau and Turner found an interaction between reward and effort although few neurons were modulated by effort alone. Despite some differences in design and analysis, taken together these studies could support a view in which SNc contains neurons that may signal the net-value of a particular behavior at the time of a cue that is instructive of the cost and benefits of acting.

By suggesting that SNc—or DA neurons in SNc specifically—does not energize actions, Varazzani et al.'s (2015) results appear at first glance to contrast with many studies implicating DA in modulating the force and speed of actions, often referred to as vigor. Considerable research has shown that blocking DA transmission, e.g., through neurotoxic lesions or drugs in the ventral striatum, reduces the selection of high effort behaviors, reduces behavioral activation, and the exertion of effort (Mai et al., 2012; Salamone and Correa, 2012). Furthermore *loss* of SNc dopaminergic neurons, as seen in Parkinson's Disease (PD), is associated with slow movements (Shiner et al., 2012). If dopamine is indeed crucial for vigor (Beierholm et al., 2013), how can we interpret the current findings, in which the activity of DA neurons in the SNc does *not* increase with effort exerted when a chosen action is executed?

We argue that the findings in PD could be consistent with the interpretation of Varazzani et al. Although PD is a disorder of motor control, patients often can generate entirely normal levels of force, but have impaired *initiation* and speed of movement (Phillips et al., 1994). Therefore, rather than modulating the amplitude of force generated, SNc DA might instead be involved in controlling the *precision* of motor commands. Indeed, PD has been considered in terms of reduced precision, leading to compensatory motor slowing (Manohar et al., 2015). Conversely, increasing motor precision would allow SNc DA to make actions faster without directly influencing the arousal or energization required to produce force. Thus, DA may play important roles in motivation in terms of choosing what effort to exert and in controlling the precision of movements when selected, but not in scaling the force exerted.

How might NA in the LC contribute to the exertion of effort? Based on consistent correlations between autonomic responses and effort, the LC-NA projection has been proposed to form a “global sympathetic system specialized in mobilization for action” (Nieuwenhuis et al., 2011). More specifically, NA could increase energetic investment or enlarge the resources available, at critical moments. It might potentially achieve these effects by increasing the excitability of neurons in the motor system. Future experiments could confirm this hypothesis by causal manipulations. In contrast to studies showing a link between DA and motivation, unfortunately, data on the effects of pharmacological manipulations of NA transmission are lacking. One recent study failed to find any effects of NA blockade on cost-benefit decisions, but did show increased response latency and omissions, as would be predicted by a functional dissociation between NA and DA (Hosking et al., 2015).

Yu and Dayan (2005) have argued that instead of energization, LC activity—and pupil dilation—is driven by *uncertainty*, since cues signifying uncertainty increase both pupil size and LC responses. In Varazzani et al.'s (2015) task, the receipt of reward was contingent on successful exertion of the imposed effort, so reward uncertainty might indeed increase with the effort required. Nonetheless, even if their findings cannot distinguish between the role of the LC in arousal or uncertainty, they suggest an important role for LC NA in the execution of actions.

Overall, Varazzani and colleagues have provided valuable insights into the neural mechanisms contributing to motivation. By achieving the considerable feat of directly comparing SNc and LC neural activity in a single study, they were able to demonstrate differential signatures of each of these neuromodulators for action selection and energization. The proposal of how these two neurotransmitters might work in tandem represents a significant step toward establishing the function of neuromodulators for cognition and also how their dysfunction may lead to motivational deficits in many neurological conditions.

## Funding statement

This research was funded by an A^*^STAR National Science Scholarship to YA, a Wellcome Trust Research Training Fellowship to SM (WT090201MA), and a BBSRC AFL Fellowship Grant to MA (BB/M013596/1).

### Conflict of interest statement

The authors declare that the research was conducted in the absence of any commercial or financial relationships that could be construed as a potential conflict of interest.
